# The Role of Gut Microbiota-Derived Trimethylamine N-Oxide in the Pathogenesis and Treatment of Mild Cognitive Impairment

**DOI:** 10.3390/ijms26031373

**Published:** 2025-02-06

**Authors:** Haihua Xie, Jia Jiang, Sihui Cao, Xuan Xu, Jingyin Zhou, Ruhan Zhang, Bo Huang, Penghui Lu, Liang Peng, Mi Liu

**Affiliations:** School of Acupuncture & Tuina and Rehabilitation, Hunan University of Chinese Medicine, Changsha 410208, China

**Keywords:** trimethylamine-N-oxide, mild cognitive impairment, gut microbiota, metabolism, brain disease, risk factor, mechanism, therapy

## Abstract

Mild cognitive impairment (MCI) represents a transitional stage between normal aging and dementia, often considered critical for dementia prevention. Despite its significance, no effective clinical treatment for MCI has yet been established. Emerging evidence has demonstrated a strong association between trimethylamine-N-oxide (TMAO), a prominent metabolite derived from the gut microbiota, and MCI, highlighting its potential as a biomarker and therapeutic target. TMAO has been implicated in increasing MCI risk through its influence on factors such as hypertension, cardiovascular disease, depression, diabetes, and stroke. Moreover, it contributes to MCI by promoting oxidative stress, disrupting the blood–brain barrier, impairing synaptic plasticity, inducing inflammation, causing mitochondrial metabolic disturbances, and facilitating abnormal protein aggregation. This review further explores therapeutic strategies targeting TMAO to mitigate MCI progression.

## 1. Introduction

Mild cognitive impairment (MCI) is a heterogeneous clinical syndrome marked by reductions in memory or other cognitive functions that do not significantly interfere with daily life and fail to align with the diagnostic criteria for dementia [1]. MCI is an intermediate state between normal aging and early dementia, with its prevalence increasing with age and lower levels of education [2]. A global epidemiological study has indicated that 15.56% of community-dwelling individuals are affected by MCI [2]. Additionally, it has been reported that 19–50% of individuals with MCI progress to dementia within three years [3]. Notably, patients with MCI retain the potential for cognitive recovery [4], with a reported reversal rate of up to 31.23% [5]. This makes the MCI stage a pivotal period for implementing preventive measures and early interventions, which are essential for delaying or preventing the onset of Alzheimer’s disease (AD) and alleviating the substantial financial and caregiving burden on families and communities. However, the precise pathogenesis and etiological factors underlying MCI remain unclear, and no effective pharmacological treatments currently exist to slow or cure the condition [6].

In recent years, the link between the gut microbiota, especially its metabolites such as trimethylamine N-oxide (TMAO), and human diseases has emerged as a research hotspot. Numerous studies have demonstrated that the gut and the brain can interact through multiple pathways. Specifically, on the one hand, the brain regulates all physiological activities in the gut. On the other hand, the gut can react to the brain through neurological, immunological, and humoral mechanisms. Given the anatomical separation and distance between the gut and the brain, the gut microbiota plays a significant role in this interaction, including activating the autonomic nervous system [7], upregulating neurotransmitters such as dopamine [8], influencing immune cells [9], and producing metabolites including TMAO [10].

TMAO has garnered considerable attention due to its strong association with cognitive function [11,12,13,14]. Buawangpong N. et al. [15] demonstrated that elevated TMAO levels independently increase the risk of MCI and suggested that plasma TMAO could serve as a predictive biomarker for MCI risk based on blood sample analysis from 233 individuals at high risk of cardiovascular disease. Xu R. et al. [16] utilized a data-driven, integrated computational approach to analyze the relationship between microbial metabolites and Alzheimer’s disease development, identifying TMAO as the metabolite most strongly correlated with AD biomarkers among 56 metabolites linked to AD pathology. However, a recent Mendelian randomization study [17] suggested no direct causal relationship between serum TMAO and its precursor substances with dementia, thereby complicating the understanding of TMAO’s role in cognitive function. The critical role of TMAO in MCI pathophysiology and its potential as a therapeutic target remain unclear.

Therefore, this review will initially investigate the changes in the gut microbiota metabolite TMAO among MCI patients. Subsequently, it will discuss how TMAO alterations may directly or indirectly influence MCI pathogenesis. Finally, therapeutic strategies targeting TMAO for managing MCI will be reviewed, emphasizing the importance of addressing TMAO as a therapeutic focus.

## 2. Origin and Excretion of TMAO

### 2.1. Origins of TMAO

**Figure 1:**
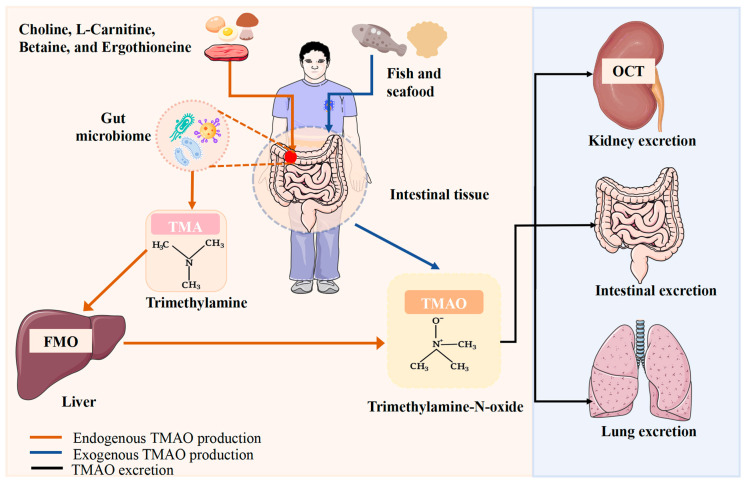
Origins and Excretion of TMAO. TMAO is absorbed directly from dietary sources through the intestines. Exogenous TMAO is subsequently produced via oxidation by the gut microbiome and the liver. The primary excretion pathways for TMAO include urine, feces, and respiration. Abbreviations: TMA, trimethylamine; TMAO, trimethylamine N-oxide; FMO, flavin-containing monooxygenase; OCT, organic cation transporter.

TMAO is a small, inert molecule with the molecular formula (CH3)3NO. It functions as a protective osmolyte, counteracting urea-induced protein denaturation and stabilizing proteins [18]. Furthermore, TMAO is closely associated with cholesterol metabolism, oxidative stress, inflammatory responses, and atherosclerosis. TMAO is derived from both exogenous and endogenous sources, as illustrated in Figure 1.

#### 2.1.1. Endogenous TMAO

Endogenous TMAO is synthesized naturally as a compound resulting from the metabolism of the gut microbiota. Following ingestion, foods abundant in choline, betaine, L-carnitine, and ergothioneine—including mushrooms, red meat, dairy products, eggs, and legumes—are metabolized into trimethylamine (TMA) by the gut microbiota, (e.g., *Firmicutes*, *Actinobacteria*, *Proteobacteria*) [19,20].

Choline is a principal precursor of TMAO and is predominantly found in animal-derived foods, particularly eggs and liver. In foods, choline exists primarily in the form of phosphatidylcholine, which is broken down during digestion by phospholipase D, releasing free choline into the circulation [19]. Within the gut, choline is metabolized by specific microorganisms to produce TMA, a process facilitated by the enzyme choline TMA lyase. This enzyme mainly exists in the intestinal microbiota, especially in certain bacteria within the *Firmicutes* and *Bacteroidetes phyla*. Additionally, choline can be metabolized into betaine via pathways involving betaine aldehyde dehydrogenase and choline dehydrogenase. Betaine, in turn, participates in the formation of TMA [19].

Betaine, another significant precursor of TMA, is primarily derived from plants and is converted to TMA by betaine reductase [21]. Similarly, L-carnitine, primarily found in animal products, undergoes transformation into TMA through the action of carnitine monooxygenase. L-Carnitine is also transformable into betaine or γ-butyrobetaine through the action of L-carnitine dehydrogenase and carnitine CoA transferase, respectively [19,21]. Both of these compounds may also contribute to TMA formation.

Ergothioneine, derived from histidine, is found in mushrooms, certain meats, and legumes. It is degraded by ergothioneine dioxygenase to produce TMA, contributing to TMAO biosynthesis [19].

The gut microbiota holds a pivotal position in TMAO biosynthesis. The metabolic capabilities of the gut microbiota vary across species, enabling them to convert precursor substances such as L-carnitine, betaine, and choline into TMA. This process involves several enzymes, including carnitine monooxygenase, choline TMA lyase, and betaine reductase. The flora involved in this process are mainly *Firmicutes* (such as *Lachnoclostridium*, *Clostridium hathewayi*, *Clostridium sporogenes*, and *Clostridium asparagiformis*) and *Proteobacteria* (including *Escherichia coli MS 200-1*, *Escherichia fergusoni*, *Proteus penneri*, *Edwardsiella tarda*, and *Desulfovibrio desulfuricans*) [22,23]. Dietary habits influence the composition and functionality of the gut microbiota. Diets rich in choline and L-carnitine increase the abundance of TMA-producing microorganisms, thereby elevating plasma TMAO levels.

Following its formation in the intestine, TMA is passively diffused into the portal venous circulatory system. In the liver, it is then metabolized to TMAO by flavin-containing monooxygenases (FMOs), predominantly FMO3 and FMO1 [24]. FMO3 is the primary enzyme responsible for this transformation, exhibiting ten-fold higher specific activity in the liver compared to FMO1.

#### 2.1.2. Exogenous TMAO

In addition to endogenous biosynthesis, TMAO can also be obtained directly from dietary sources, particularly fish and seafood. TMAO present in such foods is absorbed directly into the bloodstream by the intestine, significantly contributing to plasma TMAO levels.

### 2.2. Excretion of TMAO

Urinary excretion is the primary pathway for the elimination of TMAO from the systemic circulation. Approximately 95% of trimethylamine undergoes oxidation to form TMAO, which is subsequently excreted in urine [11,25]. This process is predominantly completed within 24 h and involves the action of multiple transport proteins, among which the organic cation transporter (OCT) plays a pivotal role. The transporters OCT1, OCT2, and OCT3 are involved in TMAO translocation, with OCT1 and OCT2 being particularly critical for regulating TMAO kinetics in murine models. Human OCT2 demonstrates the capacity to absorb TMAO; however, its role in renal tubular secretion is minimal under normal physiological conditions. Elevated TMAO levels observed in OCT1/2 knockout mice compared to wild-type controls further highlight the importance of these transporters in TMAO excretion.

In addition to urinary excretion, other pathways for TMAO elimination include fecal excretion (approximately 4%) and exhalation (less than 1%). For directly ingested TMAO, approximately 50% is absorbed into the systemic circulation and excreted in urine, while the remaining 50% is reduced back to TMA in the gut by the enzyme TMAO reductase [19].

## 3. Abnormal Levels of TMAO in MCI

The majority of clinical and preclinical studies suggest that TMAO levels are elevated in association with cognitive impairment. Several researchers have reported that patients with MCI exhibit higher TMAO levels compared to cognitively healthy individuals [26,27]. Zhu Zhaozhang et al. [28] demonstrated that the MCI group displayed a higher prevalence of elevated TMAO levels compared to the non-MCI group. Additionally, they observed a negative correlation between the Montreal Cognitive Assessment (MoCA) scores and factors such as dialysis vintage, diabetes, and TMAO levels. They concluded that elevated TMAO independently increases the risk of MCI.

Li D. et al. [13] identified a link between elevated TMAO levels and impaired cognitive function in murine models. Their study revealed that plasma TMAO levels were significantly higher in aging mice, and exogenous TMAO supplementation led to an increase in senescent cells within the hippocampal cornu ammonis 3 region. This was accompanied by cognitive impairments in the experimental group, as opposed to the control group.

From a mechanistic perspective, patients with Alzheimer’s disease and MCI have been found to exhibit higher cerebrospinal fluid (CSF) TMAO levels than cognitively normal older individuals. Elevated TMAO levels in CSF are associated with pathological biomarkers, including phosphorylated tau protein, the tau/amyloid beta (Aβ)-42 ratio, and markers of neuronal degeneration such as total tau protein and neurofilament light chain [26]. Similar findings have been observed in patients with AD [29], heart failure [30], stroke [31], Parkinson’s disease [32], and dementia [26].

Conversely, some researchers, such as Yuan Wei [33], have reported lower serum TMAO levels in patients with MCI compared to cognitively normal individuals. Similarly, a study on Parkinson’s disease found that TMAO levels in patients were lower than those in the healthy control group [32]. These discrepancies may be attributed to factors such as population heterogeneity, racial differences, and variability in control groups.

In summary, cognitive impairment is generally accompanied by elevated TMAO levels (Table 1), although variations in the findings highlight the need for further investigation into the underlying factors contributing to these discrepancies.

## 4. TMAO and MCI Risk Factors

As a metabolite of the gut microbiota, TMAO plays a critical role in various risk factors associated with MCI, including hypertension, cardiovascular disease, depression, stroke, diabetes, and other diseases. By influencing these conditions, TMAO indirectly contributes to an increased incidence of MCI (Graphical Abstract).

### 4.1. TMAO and Hypertension

Hypertension is a significant risk factor for MCI. A cross-sectional study conducted by Jia L. et al., involving 46,011 older individuals aged 60 and above in China, identified hypertension as a prominent risk factor for MCI. Specifically, individuals with a history of hypertension exhibited a 1.62-fold higher risk of developing MCI compared to those without such a history [36]. A meta-analysis incorporating data from 49 studies further suggested that cardiovascular disease is a risk factor for MCI, with the risk of developing MCI being 1.731 times higher in individuals with a history of cardiovascular disease compared to those without [37].

The association between TMAO and hypertension has garnered considerable attention. Jiang et al. observed a substantial elevation in plasma TMAO levels in hypertensive patients compared to normotensive individuals [38]. Meta-analyses have further confirmed a strong positive correlation between TMAO levels and hypertension, with evidence suggesting a dose-dependent relationship. For every 5–10 mmol/L increase in TMAO levels, the risk of hypertension rises by 9% to 20% [39].

Animal studies have further elucidated this relationship, demonstrating that inhibiting the production of gut microbiota-derived TMAO alleviates hypertension in murine models [40]. Similar findings have been corroborated by clinical trials [41,42]. Additionally, Wang H. et al. utilized Mendelian randomization techniques to establish a causal link between TMAO levels and hypertension. Their findings indicated that a one-unit increase in TMAO corresponds to a significant rise in systolic blood pressure [43,44].

### 4.2. TMAO and Stroke

Numerous studies have identified stroke as a significant cause of MCI [45,46,47,48]. A cross-sectional study by Cong et al. [48], involving 5068 older residents in China, reported that stroke patients were 1.43 times more likely to develop MCI compared to those without a history of stroke. Similarly, a one-year follow-up study conducted by Ihle-Hansen H. et al. [49] revealed that among 105 first-time stroke patients without prior cognitive impairment, 69 developed MCI.

Clinical studies have consistently shown significantly elevated TMAO levels in stroke patients compared to control groups [50,51,52]. A nested case–control study analyzing data from 16,113 participants found that higher TMAO levels are associated with an increased risk of stroke [53]. Specifically, a 1 μmol/L increase in the TMAO concentration correlates with a 12.1% heightened risk of acute ischemic stroke [54]. This association may be mediated through mechanisms such as inflammasome activation [55], glial cell proliferation [56], upregulation of mRNA expression [57], arteriosclerosis [58], and thrombosis [59].

TMAO is closely linked to stroke in multiple dimensions. On the one hand, it serves as a predictor of stroke outcomes, including vascular disease recurrence [50], poor functional recovery [60], neurological deterioration [61], and mortality [60]. On the other hand, mechanistic studies have indicated that regulating TMAO levels through targeted interventions may ameliorate the clinical symptoms of stroke, thereby providing indirect evidence of the close relationship between TMAO and stroke [62,63,64].

### 4.3. TMAO and Depression

There is a well-documented association between depressive symptoms and cognitive decline. Some researchers have proposed that depression acts as a predisposing factor for subsequent cognitive impairment, eventually leading to the development of MCI [65,66]. This correlation varies depending on the severity of depression [67]. A cross-sectional study conducted by Yang Lujing et al. [68], involving 572 older Chinese patients with MCI, identified depression as a risk factor for frailty in older individuals with MCI. Moreover, a meta-analysis encompassing 13 studies demonstrated that depression can predict the onset of MCI, particularly amnestic MCI. Specifically, individuals exhibiting depressive symptoms were found to have a 1.49-fold increased risk of developing MCI compared to those with normal cognitive function [67].

TMAO has also been linked to depression. In an animal study [69], Li X. et al. observed elevated plasma TMAO levels in rats from both the middle cerebral artery occlusion model group and the post-stroke depression group, compared to the control group. Clinical studies have similarly confirmed that TMAO levels in patients with depression exceed those in individuals without depression and exhibit a significant positive correlation with the severity of depressive symptoms [70]. Interestingly, Zheng P. et al. [71], through the analysis of urinary metabolites, reported a reduction in TMAO levels in patients with severe depression compared to healthy controls. As discussed earlier, TMAO is primarily excreted via the kidneys in urine. This abnormality in excretion may contribute to the elevated TMAO levels observed in patients with depression, albeit indirectly.

### 4.4. TMAO and Cardiovascular Disease

A robust correlation exists between cardiovascular disease and cognitive decline. High-quality evidence from a meta-analysis of 49 studies indicates that cardiovascular disease is a risk factor for MCI [37]. The risk of developing MCI in individuals with cardiovascular disease was 1.671 times greater than in those without a history of the condition.

Research has established a significant association between TMAO levels and cardiovascular disease. Tang et al. [72] conducted a large-scale clinical study involving 4007 patients with cardiovascular disease, 74% of whom were diagnosed with coronary heart disease. Over a three-year follow-up period, elevated baseline TMAO levels were found to be significantly correlated with an increased incidence of major adverse cardiovascular events. Subgroup analyses of low-risk populations also demonstrated that higher TMAO levels serve as a reliable indicator of an elevated risk of cardiovascular incidents. Meta-analyses further corroborate a strong positive relationship between TMAO levels and the risk of coronary heart disease [73], with this risk varying according to dosage. For each 1 μmol/L increase in the plasma TMAO concentration among coronary heart disease patients, the likelihood of poor prognosis rises by 1.10-fold. The underlying reason for this may be related to TMAO’s role in accelerating the development of atherosclerosis and increasing the risk of thrombosis [74].

### 4.5. TMAO and Diabetes

Diabetes is a critical risk factor for the development of MCI [75,76]. A meta-analysis study by Li J.Q. et al. [77], involving 14,821 participants, reported that the risk of developing MCI in individuals with diabetes-MCI was 1.7 times greater than in those with only MCI. Its influence on MCI is primarily mediated through insulin resistance. Yang C. et al. used nuclear magnetic resonance technology to analyze the relationship between insulin resistance and MCI, as well as type 2 diabetes mellitus (T2DM). They found that the hippocampal volume was the smallest in the high insulin resistance group among patients with T2DM and MCI [78], suggesting that insulin resistance may be a cause of cognitive decline in T2DM patients.

A substantial body of evidence supports a strong association between TMAO and diabetes [79]. In patients with type 2 diabetes, gut microbiota homeostasis is disrupted, leading to compromised intestinal barrier integrity and increased epithelial permeability. These changes collectively impact the biosynthesis and absorption of TMAO [80]. Diabetic individuals exhibit significantly elevated plasma TMAO levels compared to the general population, and these levels are positively correlated with metabolic parameters such as blood glucose and lipid concentrations. A Mendelian randomization study has suggested that T2DM contributes to increased TMAO levels [81]. The mechanisms by which TMAO influences diabetes may be related to impaired insulin responsiveness, resulting in insulin resistance and elevated blood glucose levels [82,83].

### 4.6. TMAO and Other Diseases

Studies have shown that TMAO concentrations are significantly higher in patients with chronic kidney disease (CKD) [84]. Interestingly, Kelly D.M. et al. [85] proposed that the level of albuminuria is significantly associated with the occurrence of MCI, and CKD can significantly predict the incidence of vascular dementia, suggesting that CKD is a risk factor for cognitive decline. The underlying mechanisms may be related to the promotion of plasma phosphorylated tau protein levels [86] and the induction of vascular damage [87]. Therefore, TMAO may also indirectly contribute to MCI by facilitating the development of CKD.

In addition, cancer is also closely associated with TMAO and with the development of MCI. Miller S.M. et al. [88] found metastatic renal cell carcinoma to be a risk factor for MCI, with a risk ratio of 8.52. Similar risk factors include pre-menopausal bilateral oophorectomy [89] and some gastrointestinal disorders.

Furthermore, an increased risk of MCI is associated with fewer bowel movements [90] and inflammatory bowel disease [91]. Abnormal changes in TMAO levels are related to the onset of diseases. For instance, Wilson A. et al. [92] conducted a cross-sectional observational study and found that plasma TMAO concentrations were lower in patients with inflammatory bowel disease compared to non-diseased individuals.

## 5. The Role of TMAO in the Pathogenesis of MCI

TMAO, a critical metabolite derived from the gut microbiota, exhibits a significant positive association with MCI. Notably, TMAO contributes to a range of biological dysfunctions, including oxidative stress, disruption of the blood–brain barrier, decreased synaptic plasticity, inflammation, mitochondrial dysfunction, and abnormal protein aggregation. These dysfunctions are central to the pathogenesis of MCI. This section explores the role of TMAO in the development of MCI through the aforementioned mechanisms.

### 5.1. TMAO Promotes Oxidative Stress

Oxidative stress in the brain is a pivotal factor in the pathophysiology and progression of MCI. This condition arises from an imbalance between the production of free radicals and the antioxidant system’s capacity to neutralize them, leading to the oxidative degradation of proteins, lipids, and other cellular components. Oxidative damage is typically assessed using indices such as protein oxidation markers and lipid peroxidation levels.

The existing literature underscores the crucial role of oxidative stress in the pathogenesis of MCI [93,94], including the oxidation of ubiquitin C-terminal hydrolase [95], the lipid peroxidation product 4-hydroxy-2-nonenal (HNE) [96], damage to the phosphoinositide 3-kinase/protein kinase B/mammalian target of rapamycin (PI3K/Akt/mTOR) pathway [97], oxidative DNA damage [98], locus coeruleus oxidative stress [99], and protein oxidation of enzymes such as glutamine synthetase (GLUL), peptidyl-prolyl cis/trans isomerase 1 (PIN1), alpha-enolase (ENO1), and pyruvate kinase M2 (PKM2) in the hippocampus [100].

**Figure 2:**
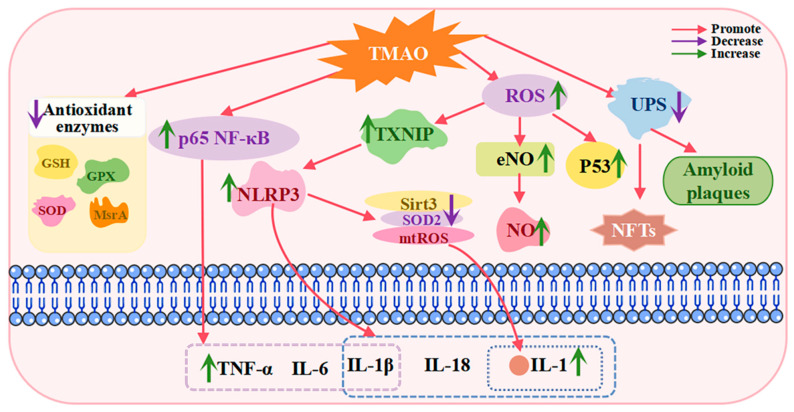
Contributions of TMAO to the pathogenesis of MCI. TMAO potentially contributes to the pathogenesis of MCI by promoting oxidative stress, neuroinflammation, and abnormal protein accumulation. TMAO induces oxidative stress by enhancing the production of reactive oxygen species (ROS) and reducing antioxidant activity. It also triggers neuroinflammation by activating NF-κβ and the NLRP3 inflammasome. Furthermore, TMAO exacerbates the formation of amyloid plaques and neurofibrillary tangles by impairing the intracellular ubiquitin–proteasome system. Abbreviations: TMAO, trimethylamine N-oxide; GSH, glutathione; GPX, glutathione peroxidase; SOD, superoxide dismutase; MsrA, methionine sulfoxide reductase A; NF-κB, nuclear factor kappa B; NLRP3, NOD-like receptor family pyrin domain containing 3; Sirt3, sirtuin 3; mtROS, mitochondrial reactive oxygen species; IL, interleukin; TXNIP, thioredoxin-interacting protein; NFTs, neurofibrillary tangles.

As a metabolite derived from the gut microbiota, elevated TMAO levels have been shown to exacerbate oxidative stress (Figure 2), thereby contributing to the pathophysiological changes observed in MCI.

Research has demonstrated that TMAO disrupts intracellular redox balance by promoting the generation of reactive oxygen species (ROS) while simultaneously reducing antioxidant activity [34]. TMAO has been shown to accelerate brain aging and cognitive decline by inducing neuronal senescence and exacerbating neuroinflammation and oxidative stress [101]. Excessive ROS production results in DNA damage, lipid peroxidation, and protein oxidation, which collectively trigger cellular apoptosis and cognitive dysfunction. In patients with MCI, elevated levels of the pro-apoptotic protein P53 and the covalent modification of the lipid peroxidation product 4-HNE exacerbate neuronal loss, thereby accelerating cognitive decline [96].

Elevated plasma TMAO levels activate the ROS/thioredoxin interacting protein (TXNIP)/NOD-like receptor family protein-3 inflammasome (NLRP3) signaling pathway, which increases the production of inflammatory agents such as interleukin-1 (IL-1) and interleukin-18 (IL-18). This process suppresses endothelial nitric oxide synthase (eNOS) and nitric oxide (NO) production, intensifying oxidative stress and inflammation in the brain. Consequently, neuronal growth is impaired, and apoptosis is accelerated [102].

TMAO also weakens cellular antioxidant defenses by impairing the expression and activity of antioxidant enzymes, rendering cells more vulnerable to oxidative stress. Recent studies have revealed that TMAO suppresses the activity of superoxide dismutase (SOD) and glutathione peroxidase (GPX) in a vascular dementia rat model [103]. Experimental evidence further indicates that elevated circulating TMAO levels may reduce antioxidant enzyme activity in the hippocampus, increase sensitivity to oxidative stress, and exacerbate postoperative neuroinflammation and cognitive decline in aged rats [104].

Glutathione (GSH), a key antioxidant responsible for maintaining intracellular redox homeostasis, plays a critical role in preserving the physiological function of all cells. Zhang Wenbo et al. [29] reported that exogenous supplementation of TMAO reduces GSH levels in the brain. Similarly, a study by Li D. et al. [13] demonstrated that mice exposed to high TMAO levels exhibited significantly reduced total SOD activity and elevated hydrogen peroxide levels in the hippocampus compared to control groups. These findings suggest that TMAO elevates oxidative stress in the hippocampus, contributing to cognitive decline.

Zhao et al. [105] observed that rats in the high TMAO group exhibited more severe cognitive dysfunction following sevoflurane treatment compared to the control group. Additionally, the expression of methionine sulfoxide reductase A (MsrA), a key antioxidant enzyme that repairs methionine residues damaged by oxidative stress, was markedly reduced in the hippocampus of high TMAO rats. MsrA protects proteins from oxidative damage, and its downregulation supports the hypothesis that TMAO-induced cognitive decline is mediated through the suppression of hippocampal antioxidant enzyme expression. This exacerbates oxidative stress, thereby intensifying cognitive dysfunction in aged rats.

### 5.2. TMAO Disrupts the Blood–Brain Barrier (BBB) and Reduces Synaptic Plasticity

**Figure 3:**
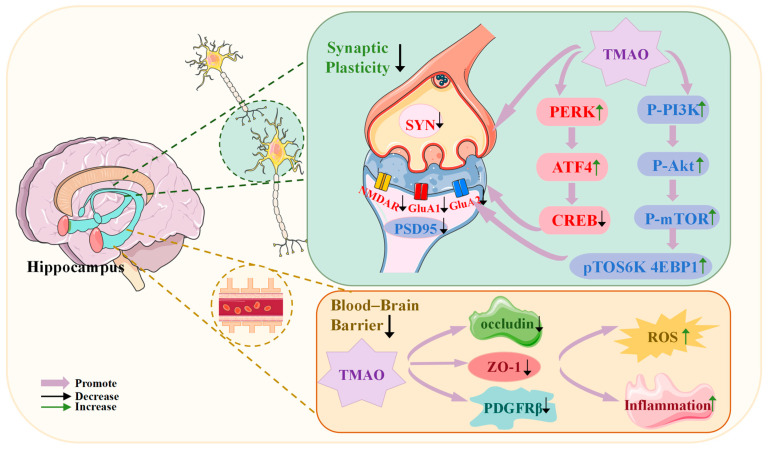
Effects of TMAO on the blood–brain barrier and synaptic plasticity. TMAO impairs the structural integrity and function of the blood–brain barrier (BBB) and reduces synaptic plasticity, contributing to the pathogenesis of MCI. It reduces hippocampal synaptic plasticity by activating the PI3K/Akt/mTOR and PERK signaling pathways. Simultaneously, TMAO disrupts the BBB, facilitating the accumulation of neurotoxic molecules in the brain and inducing oxidative stress and neuroinflammation. Abbreviations: SYN, synaptophysin; NMDAR, N-methyl-D-aspartate receptor; GluA1, glutamate receptor ionotropic AMPA 1; GluN2A, glutamate receptor ionotropic NMDA 2A; PSD95, postsynaptic density protein 95; PERK, protein kinase R-like endoplasmic reticulum kinase; ATF4, activating transcription factor 4; CREB, cAMP response element-binding protein; p-PI3K, phosphorylated phosphoinositide 3-kinase; p-Akt, phosphorylated Akt protein; p-mTOR, phosphorylated mammalian target of rapamycin; ZO-1, zonula occludens-1; PDGFRβ, platelet-derived growth factor receptor beta.

The BBB is a semipermeable structure composed of brain endothelial cells, pericytes, the basement membrane, and astrocytes. The endothelial cells are interconnected by tight junctions formed by claudins, occludin, and other tight junction proteins, which create a high-resistance cellular barrier. Disruption of these tight and adhesive connections, along with the enzymatic degradation of the capillary basement membrane, can cause physical damage to the BBB (Figure 3). Zhang Wenbo et al. [29] found that TMAO induces a reduction in the expression of zonula occludens-1 (ZO-1) and occludin in the hippocampus, leading to compromised BBB integrity. Furthermore, a decrease in the expression of the pericyte marker platelet-derived growth factor receptor beta (PDGFRβ) results in impaired BBB function, which subsequently contributes to cognitive deficits. BBB injury often leads to the accumulation of various molecules and neurotoxic products in the brain, such as immunoglobulins, albumin, and iron from hemoglobin, exacerbating neuroinflammation and generating neurotoxic reactive oxygen species. However, recent research has suggested that physiological doses of TMAO may positively regulate BBB integrity by inducing annexin-A1 [106], indicating that the relationship between TMAO and the BBB may be dose-dependent.

Synaptic plasticity, which underpins learning and memory, is also affected by TMAO, influencing cognitive function (Figure 3). Specifically, TMAO can impair hippocampal synaptic plasticity via the PI3K/Akt/mTOR signaling pathway [97]. The hippocampus, a brain region critical for memory and learning, shows a reduction in synaptic plasticity, which is directly linked to cognitive dysfunction. The mTOR signaling pathway regulates the synthesis of proteins involved in learning and memory [107]. Tramutola et al. proposed that the PI3K/Akt/mTOR pathway is overactivated in the inferior parietal lobule [97]. After TMAO intervention, the PI3K/Akt/mTOR pathway is significantly activated, with an increase in the expression of p-PI3K, p-Akt, and p-mTOR [108], as well as an elevation in the mTOR downstream targets Ribosomal protein S6 kinase beta-1 (p70S6K) and eIF4E-binding protein 1 (4EBP1) [97,109].

Additionally, TMAO has been found to impair synaptic plasticity by promoting endoplasmic reticulum (ER) stress. Long-term potentiation (LTP), a form of synaptic plasticity widely recognized as the neurobiological basis of learning and memory, is a predictor of long-term memory function [110]. TMAO reduces the expression of postsynaptic glutamatergic receptor subunits, including α-amino-3-hydroxy-5-methyl-4-isoxazole-propionic acid receptor subunits (GluA1 and GluA2) and N-methyl-D-aspartate receptor subunits (GluN2A) [101], and downregulates the expression of synaptic plasticity-related proteins such as synaptophysin, N-methyl-D-aspartate receptor, and postsynaptic density protein-95 [13,101], resulting in a reduction in LTP expression [101].

TMAO also induces synaptic plasticity defects through the PERK signaling pathway mediated by ER stress [101]. The unfolded protein response (UPR) includes three primary pathways: Activating Transcription Factor 6 (ATF6), Protein Kinase R-like Endoplasmic Reticulum Kinase (PERK), and Inositol-requiring Enzyme 1α (IRE1α), which alleviate ER stress by enhancing ER folding capacity, reducing protein synthesis, and increasing chaperone protein expression. TMAO promotes increased PERK phosphorylation [101], subsequently enhancing the phosphorylation of eIF2α at serine 51, leading to elevated ATF4 protein levels. ATF4 then exerts a negative regulatory effect on synaptic plasticity and memory by inhibiting the phosphorylation of cAMP Response Element-dependent Transcription.

### 5.3. TMAO Induces Inflammation

Neuroinflammation is a key factor in the pathogenesis of MCI. Inflammation is a response of the nervous system to injury or infection; however, sustained inflammation can result in prolonged neuronal damage and functional impairment [111].

TMAO is capable of activating inflammatory signaling pathways (Figure 2), such as the nuclear factor kappa B (NF-κB) pathway and the NOD-like receptor family pyrin domain containing 3 (NLRP3) inflammasome. Activation of these pathways can lead to neuronal damage and cognitive decline. TMAO inhibits the Sirtuin 3/Superoxide Dismutase 2/Mitochondrial Reactive Oxygen Species (Sirt3/SOD2/mtROS) signaling pathway through activation of the NLRP3 inflammasome, resulting in the increased production of inflammatory factors, such as interleukin-1, and exacerbating cerebral vasculitis [112]. Sun et al. observed [102] that TMAO can trigger the secretion of the inflammatory cytokines IL-1β and IL-18 via the ROS/Thioredoxin-Interacting Protein (TXNIP)/NLRP3 pathway. Yue et al. reported [113] that TMAO activates the release of inflammatory cytokines induced by NLRP3, leading to a cascade of inflammatory reactions, as well as ROS production, which induces oxidative stress. Yu et al. discovered [114] that TMAO significantly increases the expression of the pro-inflammatory cytokines IL-1β, IL-6, and TNF-α by activating the p65 NF-κB pathway. In an experimental study by Meng et al. [104], aged rats administered TMAO exhibited elevated plasma TMAO levels in the week preceding and following surgery. This elevation exacerbated microglial-mediated neuroinflammation in the hippocampus and promoted ROS production, ultimately aggravating cognitive dysfunction in the laparotomy group of rats. These findings suggest a correlation between elevated circulating TMAO levels, intensified postoperative neuroinflammation, and cognitive decline in aged rats.

A potential mechanism by which TMAO triggers neuroinflammation is through the stimulation of the innate immune system. In the brain, astrocytes, which are abundant and active immune cells, can become reactive in response to immune-related stressors. TMAO has the ability to cross the blood-brain barrier and directly activate astrocytes [11], resulting in impaired cognitive abilities.

### 5.4. TMAO Affects Mitochondrial Metabolism

Mitochondria are the “energy factories” of cells, responsible for producing adenosine triphosphate (ATP) and supplying energy to cellular processes. In patients with MCI, mitochondrial dysfunction is a critical factor contributing to the insufficient neuronal energy supply and cognitive decline. Mitochondrial dysfunction is characterized by the downregulation of mitochondrial complexes (e.g., cytochrome c oxidase complex) [115], reduced electron carrier activity (such as decreased cytochrome c oxidase (COX) activity and lower cytochrome c content) [116], and diminished mitochondrial membrane potential [116].

**Figure 4:**
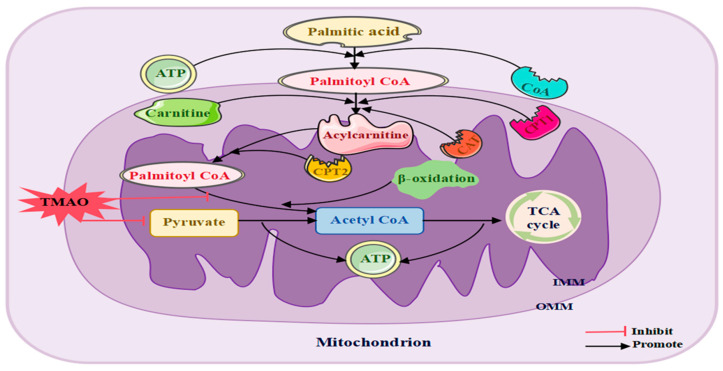
Effects of TMAO on mitochondrial metabolism. TMAO adversely affects mitochondrial metabolism, contributing to the pathogenesis of MCI. It significantly inhibits the oxidation of pyruvate and fatty acids in mitochondria, leading to energy metabolism disorders. Abbreviations: CAT, carnitine acylcarnitine translocase; CPT2, carnitine palmitoyl transferase II; TCA cycle, tricarboxylic acid cycle; IMM, inner mitochondrial membrane; OMM, outer mitochondrial membrane; ATP, adenosine triphosphate.

TMAO can affect mitochondrial function (Figure 4). TMAO significantly impedes the breakdown of pyruvate and fatty acids in cardiac mitochondria [117], potentially impairing normal mitochondrial functions, which affect cellular energy distribution and metabolic equilibrium. Additionally, TMAO may indirectly influence mitochondrial activity by disrupting mitochondrial DNA integrity [118], intensifying inflammatory responses, and disrupting epigenetic regulation, thereby disturbing mitochondrial balance [119]. Notably, in certain pathological conditions, such as a rat model of right ventricular heart failure, TMAO demonstrates protective effects on mitochondrial energy metabolism and cardiac performance [120], highlighting its dose-dependent nature.

Further research has demonstrated that TMAO exacerbates mitochondrial dysfunction by altering mitochondrial morphology. Li et al. [13] found that TMAO supplementation increased the number of senescent cells and enhanced mitochondrial damage in the hippocampal CA1 region. Mitochondrial damage manifested as swelling and deformation of mitochondria and the endoplasmic reticulum, a reduction in mitochondrial cristae, and an accumulation of lipofuscin within cells.

TMAO may also cause oxidative stress in the brain, leading to mitochondrial metabolic dysfunction. Oxidative stress is significantly increased in AD vertebral neurons, which in turn increases mitochondria autophagic degradation and ultimately induces cerebral neurological damage [121]. In vitro experiments further demonstrated that inducing lipid peroxidation in cerebral capillaries can cause mitochondrial fission and reduce mitochondrial respiration [122]. On the other hand, mitochondrial damage caused by TMAO may further trigger oxidative stress [123]. Under physiological conditions, mitochondrial metabolism produces ROS to maintain redox homeostasis. The impaired state of mitochondrial structure and function may exacerbate the production of ROS [124], which in turn causes neuronal cell death and disruption of the blood–brain barrier.

In summary, TMAO can cause morphological changes in mitochondria, further exacerbating functional impairment, and can also reduce ATP generation, triggering functional impairment. Mitochondrial metabolic dysfunction causes abnormal energy metabolism, further reducing the number of neurons and leading to functional abnormalities. This may be the cause of MCI.

### 5.5. TMAO Promotes Abnormal Protein Aggregation

The aggregation of abnormal proteins is a significant feature in the pathogenesis of MCI. AD, a progressive and incurable neurodegenerative disorder, is characterized by the accumulation of Aβ peptide and hyperphosphorylated tau protein (p-tau) in nerve cells, forming amyloid plaques and neurofibrillary tangles (NFTs), respectively. As a precursor to AD, MCI already exhibits abnormal protein aggregation [125], although to a lesser degree.

It has been established that TMAO is closely linked to the formation of amyloid plaques and neurofibrillary tangles, as well as the development of Alzheimer’s disease [26]. TMAO promotes the aggregation of abnormal proteins by interfering with the normal folding and clearance mechanisms of proteins. Proper protein folding is essential for maintaining protein structure and function, and TMAO, at supraphysiological concentrations, can disrupt this process, leading to protein misfolding and aggregation. Furthermore, TMAO exacerbates the aggregation and deposition of abnormal proteins by affecting intracellular protein degradation systems, such as the ubiquitin–proteasome system [95] (Figure 2).

Under normal physiological conditions, microglia, macrophages, and astrocytes clear secreted Aβ from the brain’s interstitial space. However, at supraphysiological concentrations, TMAO accelerates the transformation of Aβ fibers into β-lamellar conformations, promoting Aβ aggregation and stabilizing the Aβ aggregates [126,127]. Zhang Wenbo et al. [29] found that supplementing with TMAO increased the area and number of Aβ plaques in the hippocampus of APPswe/PSEN1dE9 mice, suggesting that TMAO stabilizes β-amyloid protein, leading to cognitive impairment. Experimental studies have shown that TMAO can accelerate the aggregation of β-amyloid protein [126] or α-synuclein [98] under specific conditions.

Tau, a microtubule-associated protein, can be affected by changes in its quantity, structure, and functionality, influencing its assembly. Studies indicate that TMAO enhances tau protein clustering by stabilizing both intramolecular and intermolecular hydrogen bonds, reducing the aggregation concentration required and shortening the delay in aggregation [128,129]. TMAO can also induce the formation of secondary structures in the C-terminal segment of tau and significantly enhance tau’s self-clustering and microtubule construction, leading to the formation of neurofibrillary tangles [129].

## 6. TMAO and Intervention in MCI

### 6.1. Dietary Structure

Different dietary patterns have distinct effects on MCI [130,131,132]. Aarsland et al. [133] proposed that anthocyanins are safe and effective in enhancing cognitive function and mitigating the risk of dementia in MCI patients, while an unhealthy diet increases the risk of cognitive impairment. A study of 11,157 seniors aged 65 and older in China revealed that poor adherence to a healthy diet is a primary risk factor for MCI in this demographic [134]. Various food types play different roles in the onset of MCI. A meta-analysis by García-Casares et al. [135] suggested that adherence to the Mediterranean diet model can effectively reduce the incidence of MCI, based on data from 22 studies. A cross-sectional study of 1262 older individuals [136] found that meat, eggs, and grains serve as protective factors against MCI. Modifying lifestyle and dietary habits can effectively improve cognitive function in older adults, MCI patients, and AD patients [137,138,139,140].

Scientific evidence confirms that diets rich in choline, carnitine, saturated fatty acids, and animal proteins negatively affect the growth of the gut microbiota and elevate plasma TMAO levels [58]. A recent study [109] demonstrated that TMAO, a choline metabolite, triggers learning and memory deficits in the hippocampus, along with synaptic plasticity issues, through activation of the mTOR signaling pathway. A variety of nutrients, such as dietary fiber, polyphenols, and fatty acids, play significant roles in the influence of dietary structure on TMAO.

Dietary fiber is a nutrient that is digested and absorbed in the human distal colon and significantly reduces peripheral blood TMAO levels in humans [141,142]. Notably, inulin, as well as inulin-type fructans, improved the gut microbial composition but did not significantly reduce plasma TMAO levels [143,144]. Dietary fiber includes multiple types, and identifying specific types of dietary fiber that can reduce TMAO levels is a promising direction for future research.

Polyphenol is a collective term for a group of plant-derived chemicals with strong antioxidant and anti-inflammatory properties. It plays an important role in the prevention and treatment of chronic diseases in older adults. Studies have shown that polyphenol supplementation significantly improved executive function and spatial learning ability in older individuals with MCI. Furthermore, several clinical and animal experiments have shown that polyphenols such as resveratrol and Taurisolo can significantly reduce TMAO levels in the blood [145,146].

Omega-3 fatty acids are important unsaturated fatty acids for human health. The results from an animal experiment indicate that docosahexaenoic acid, a type of omega-3 fatty acid, significantly reduced the level of TMAO in serum [147]. Some scholars have also suggested that krill oil, which is rich in omega-3 fatty acids, may increase the concentration of some TMAO precursors but has no significant effect on plasma TMAO levels [148]. Currently, there are relatively few studies in this area, and further exploration is needed.

**Figure 5:**
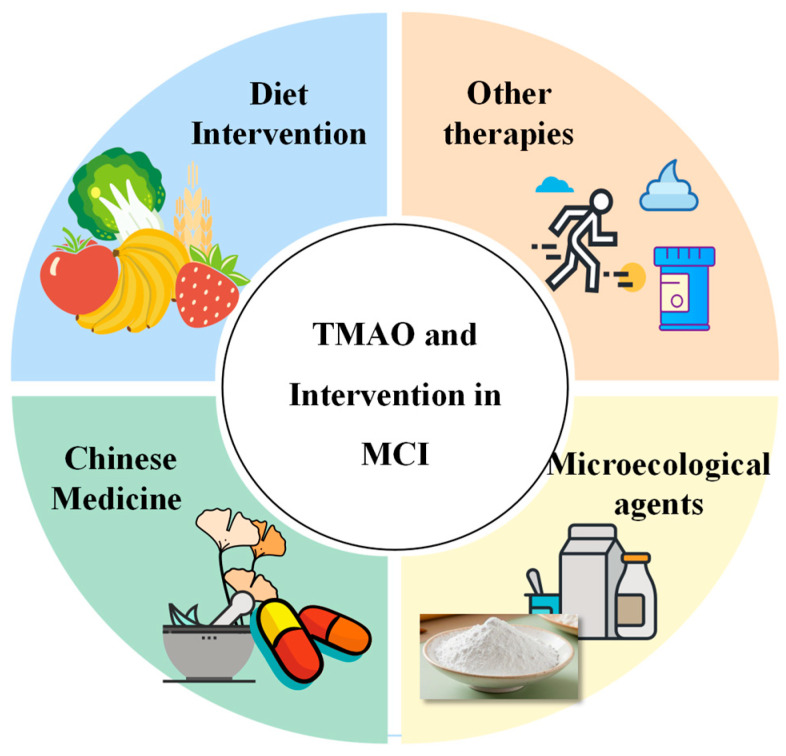
Treatment Strategy for improving MCI by affecting TMAO.

In summary, dietary structure is closely associated with plasma TMAO levels, making dietary guidance particularly crucial for MCI patients (Figure 5). Although there are currently no studies specifically designed to reduce TMAO levels through dietary control and cognitive modulation, attention should be paid to the implementation of a balanced diet in healthy individuals to prevent the potential risk of elevated TMAO levels contributing to the onset of MCI.

### 6.2. Traditional Chinese Medicine

As a primary treatment modality in traditional Chinese medicine, Chinese herbal medicine has been shown to possess therapeutic potential for MCI patients, as evidenced by multiple studies [149]. Such treatments include Yishen Granules [150], compound Chinese medicine Bushen Capsules [151], modified Guipi Decoction [152], Ginkgo biloba leaves [153,154,155], the heart and kidney regulating method [156], the kidney tonifying and stasis removing method [157], the Chinese medicine compound “Tiantai No.1” [158], and the improved Huanglian Wendan Decoction [159]. A multicenter clinical randomized controlled trial suggested that acupuncture and moxibustion, in combination with Yishen Granules, can improve cognitive function in MCI patients, with the combined treatment yielding superior results [150]. Further investigations have explored the effectiveness of Chinese medicine in modulating TMAO to treat cognitive impairment, particularly through foundational experimental studies. Zhao Jinli [160], based on animal experiments, found that curcumin counteracts TMAO-induced pyroptosis and mitochondrial dysfunction in human umbilical vein endothelial cells by upregulating the expression of the mitochondrial gene ubiquinol cytochrome c reductase core protein 1. Liu J. et al., through behavioral testing, liquid chromatography–mass spectrometry, transmission electron microscopy, Nissl staining, and Western blot and immunohistochemical staining, demonstrated that ZeXieYin Formula could ameliorate TMAO-induced cognitive decline in mice, repair synaptic damage, and regulate synaptic proteins and the mTOR pathway, thus presenting itself as a promising novel therapeutic approach for treating TMAO-related cognitive impairments [161].

### 6.3. Microecological Agents

Microecological agents are biological agents or products that regulate and improve the microecological balance of the human body. They can be categorized as probiotics, prebiotics, and synbiotics.

Probiotics are living microorganisms that regulate the gut microbiota, optimize the gut environment, maintain homeostasis, promote nutrient absorption, enhance immunity, and ultimately improve human health [162]. Recent studies [163,164,165,166] have reported notable improvements in cognitive abilities following probiotic treatments, which are linked to alterations in the gut microbiota. These results suggest a potential beneficial effect of probiotics on cognitive abilities in older individuals with MCI. On the one hand, probiotics can effectively reduce the population of specific microbial species capable of producing TMA by optimizing the composition of the gut microbiota, thereby inhibiting the production of TMA in the intestine. Mitsuharu Matsumoto et al. [167] found that *Lactobacillus* LKM512, compared to a placebo, modulated the gut microbiota in healthy subjects, reducing the abundance of TMA-producing bacteria and consequently lowering TMA levels in the human intestine. Chen S. et al. [168] observed a more pronounced decrease in plasma TMAO levels in the probiotic group compared to the control group following intervention, suggesting that probiotic supplementation may modify the composition of the gut microbiota, potentially reducing the proportion of microbiota responsible for TMA production.

On the other hand, probiotics have also demonstrated the ability to directly degrade pre-existing TMA in the intestine or facilitate its conversion into other harmless or beneficial substances [169], further reducing the accumulation of TMA in the gut. An animal experiment [170] indicated that *Enterobacter aerogenes* ZDY01 could reduce serum TMAO and cecal TMA levels in choline-loaded mice. The study proposed that ZDY01 decreased the relative abundance of *Clostridium* and *Acinetobacter*, both of which produce TMA-lyase, suggesting that ZDY01 may degrade TMA. Additionally, Enterobacteriaceae contain pathways for converting TMA into dimethylamine and methylamine. A clinical trial reported similar findings. Gian Carlo Tenore et al. [171] noted a significant reduction in plasma TMAO levels among the test group treated with *Lactobacillus rhamnosus* LRH11 and *Lactobacillus plantarum* SGL07, in contrast to the control group. The study proposed that probiotic-fermented apple puree, rich in polyphenols, may regulate the gut microbiota and reduce the proportion of TMA-producing microbiota. Furthermore, in serum, TMAO and polyphenols might be involved in redox reactions, leading to the production of TMA. Most existing studies have affirmed the application of probiotics in improving TMAO levels in cardiovascular diseases and reducing MCI risk. However, no studies have yet demonstrated a direct correlation between probiotic consumption, TMAO levels, and MCI outcomes in patients, underscoring the need for further research to elucidate these potential associations.

Prebiotics are substances that can be selectively utilized by host microorganisms and converted into compounds beneficial to host health. Studies have shown that prebiotics such as xylooligosaccharide and galacto-oligosaccharides improved the composition of the intestinal microbiota [172,173]. Resveratrol [145], dietary fiber, and functional oligosaccharides can modulate the level of TMAO, while inulin supplementation does not seem to have a significant effect on TMAO [174].

Synbiotics, also known as synergistics, are mixtures of probiotics and prebiotics. Dahl W.J. et al. [175] administered a high-protein diet to healthy older women, with the synbiotic group receiving additional synbiotic supplements on this basis. The results indicated that the synbiotic supplements did not seem to reduce TMAO levels.

### 6.4. Other Therapies

Antibiotics may have a positive effect on MCI patients. A study has shown no significant changes in several blood biomarkers of MCI risk in patients with type 2 diabetes treated with topical periodontal antibiotics [176]. In contrast, another case report suggests that the symptoms of an older adult male with MCI completely disappeared after antibiotic treatment. The impact of antibiotics on TMAO is also not yet clear. Stremmel W. et al. [177] found that the antibiotic rifaximin can significantly reduce plasma TMAO levels, whereas Macpherson M.E. et al. [178] did not observe any effect of rifaximin on TMAO.

Western drug therapy also has a positive effect on MCI patients, such as vitamin B, vitamin D, and Lecanemab [179,180]. Some Western drugs can effectively reduce the level of TMAO. Obeid R. et al. [181] suggested that vitamin B combined with vitamin D treatment can significantly reduce the plasma level of TMAO. Kuka J. et al. found that meldonium treatment can inhibit the intestinal flora and reduce the plasma level of TMAO through an animal experiment. Other drugs with similar effects include ranitidine and finasteride [182].

Fecal Microbiota Transplantation (FMT) is a treatment that reconstructs the intestinal microbiota. The existing literature has demonstrated the effectiveness of FMT for MCI from multiple perspectives. Specifically, transplanting the fecal microbiome from mice with MCI into wild-type mice can induce a decline in their cognitive function [183]. On the other hand, FMT suppressed cognitive dysfunction induced by chronic cerebral underperfusion in rats [184] and improved cognitive function in patients with MCI [185]. Scholars have observed that FMT can lead to changes in TMAO in traumatic brain injury [186], Crohn’s disease [187], and CKD [188], but there are no significant changes in metabolic syndrome and atherosclerosis [189,190]. Whether FMT can affect TMAO levels in MCI patients requires further investigation.

Lifestyle guidance, such as exercise and sleep intervention, can significantly improve the cognitive function of patients with MCI [191,192]. Erickson M.L. et al. [193] analyzed the role of exercise combined with dietary intervention in obese adults in a clinical study, proposing that exercise combined with a low-calorie diet can significantly reduce plasma TMAO levels. Baptista L.C. et al. [194] suggested that exercise combined with resveratrol intervention in older individuals seems to have no effect on TMAO levels. In addition to this, it is not clear whether this changes in patients with MCI.

## 7. Conclusions and Future Prospects

MCI represents a critical stage for interventions aimed at delaying the progression to dementia, with its pathogenesis closely linked to abnormal alterations in the brain–gut axis. TMAO, a metabolite produced by the gut microbiota, has been under investigation for its role in cognitive function. The findings of this review indicate that, with the onset of MCI, TMAO levels increase in patients, leading to metabolic disturbances that may contribute to the onset and high incidence of MCI. However, most of the mechanism-related literature reviewed in this article is based on preclinical studies, with fewer exploratory studies conducted in humans. Future research should focus on investigating the specific processes linking changes in TMAO levels to the emergence of MCI in clinical settings. Furthermore, dietary modifications, microecological agents, Western drugs, FMT, lifestyle guidance, and Chinese medicine treatments have shown potential therapeutic effects in improving cognitive function in MCI. Targeted interventions aimed at TMAO may offer a novel approach for treating MCI, but their safety and underlying mechanisms remain inconclusive. Future studies should prioritize two key directions: first, conducting large-scale, high-quality clinical trials to evaluate the efficacy and mechanisms of TMAO-targeted interventions; and second, exploring the interrelationships between TMAO and other biomarkers to establish more precise predictive models for MCI. Such models would also provide a scientific foundation for individualized treatment and early intervention.

## Figures and Tables

**Table 1:** Changes in TMAO levels in different studies.

Reference	Sample	Research Subjects	TMAO Levels	Consequence
Xu N., 2022 [27]	74 /150	MCI in Chinese type 2 diabetes mellitus /healthy controls	14.16 (11.28, 18.44) /5.10 (4.48, 6.06)	Serum TMAO Increase
Vogt N.M., 2018 [26]	35 /335	MCI/cognitively unimpaired individuals	2.1 ± 1.4 /1.3 ± 1.5	Cerebrospinal fluid TMAO Increase
Li D., 2018 [13]	168 /118 /141	Young adults /middle-aged adults /older adults	2.85 ± 3.10 /4.42 ± 4.39 /9.83 ± 10.63	Plasma TMAO Increase
Zhu Z.Z., 2019 [28]	50 /58	MCI in maintenance hemodialysis (MHD) /non-MCI in MHD	196.4 ± 41.2 /109.9 ± 61.7	Serum TMAO Increase
Yuan W., 2023 [33]	112 /312	MCI/healthy controls	0.74 (0.48,1.12) /0.82 (0.55,1.32)	Serum TMAO Decrease
Li T., 2017 [34]	18 /18	Old rats/young rats	14.30 ± 1.52 /6.41 ± 1.27	Plasma TMAO Increase
Brunt V.E., 2021 [11]	22/ 103	Young adults /middle-aged and older adults	Unavailable	Plasma TMAO Increase
Brunt V.E., 2021 [11]	9/ 14	Young mice /older mice	Unavailable	Plasma TMAO Increase
He W., 2020, [35]	135/ 316	Physical and cognitive frail older adults /nonfrail older adults	4.0 (2.8–7.0) /3.2 (2.1–5.0)	Plasma TMAO Increase

TMAO, trimethylamine N-oxide; MCI, mild cognitive impairment; MHD, maintenance hemodialysis.

## Data Availability

Not applicable.
